# Reliability of Panoramic Ultrasound in Assessing Rectus Femoris Size, Shape, and Brightness: An Inter-Examiner Study

**DOI:** 10.3390/bioengineering11010082

**Published:** 2024-01-15

**Authors:** Jorge Buffet-García, Gustavo Plaza-Manzano, Umut Varol, Marta Ríos-León, María José Díaz-Arribas, Javier Álvarez-González, Sandra Sánchez-Jorge, Juan Antonio Valera-Calero

**Affiliations:** 1 Department of Physiotherapy, Faculty of Health, Universidad Francisco de Vitoria, 28223 Pozuelo de Alarcón, Spain; j.buffet.prof@ufv.es (J.B.-G.); j.alvarezglez.prof@ufv.es (J.Á.-G.); 2 Department of Radiology, Rehabilitation and Physiotherapy, Universidad Complutense de Madrid, 28040 Madrid, Spain; gusplaza@ucm.es (G.P.-M.); mjdiazar@med.ucm.es (M.J.D.-A.); juavaler@ucm.es (J.A.V.-C.); 3 Grupo InPhysio, Instituto de Investigación Sanitaria del Hospital Clínico San Carlos, 28040 Madrid, Spain; 4 Escuela Internacional de Doctorado, Universidad Rey Juan Carlos, 28922 Alcorcón, Spain; au.varol.2022@alumnos.urjc.es; 5 Sensorimotor Function Group, Hospital Nacional de Parapléjicos, Servicio de Salud de Castilla-La Mancha (SESCAM), 45004 Toledo, Spain; mriosl@sescam.jccm.es; 6 Instituto de Investigación Sanitaria de Castilla-La Mancha (IDISCAM), 45004 Toledo, Spain

**Keywords:** panoramic ultrasound, diagnostic accuracy, ultrasound imaging, quadriceps

## Abstract

Extended field-of-view ultrasound (US) imaging, also known as panoramic US, represents a technical advance that allows for complete visualization of large musculoskeletal structures, which are often limited in conventional 2D US images. Currently, there is no evidence examining whether the experience of examiners influences muscle shape deformations that may arise during the glide of the transducer in panoramic US acquisition. As no studies using panoramic US have analyzed whether two examiners with differing levels of experience might obtain varying scores in size, shape, or brightness during the US assessment of the rectus femoris muscle, our aim was to analyze the inter-examiner reliability of panoramic US imaging acquisition in determining muscle size, shape, and brightness between two examiners. Additionally, we sought to investigate whether the examiners’ experience plays a significant role in muscle deformations during imaging acquisition by assessing score differences. Shape (circularity, aspect ratio, and roundness), size (cross-sectional area and perimeter), and brightness (mean echo intensity) were analyzed in 39 volunteers. Intraclass correlation coefficients (ICCs), standard error of measurements (SEM), minimal detectable changes (MDC), and coefficient of absolute errors (CAE%) were calculated. All parameters evaluated showed no significant differences between the two examiners (*p* > 0.05). Panoramic US proved to be reliable, regardless of examiner experience, as no deformations were observed. Further research is needed to corroborate the validity of panoramic US by comparing this method with gold standard techniques.

## 1. Introduction

Ultrasound (US) imaging is a popular tool across various disciplines because it offers real-time data without the use of ionizing radiation and is a fast and cost-effective alternative to other imaging techniques such as Magnetic Resonance Imaging (MRI) or Computed Tomography (CT) [1]. In addition, it can be used as a rehabilitation tool, since is used to assist the patients in the correct performance and learning of motor control and pelvic floor exercises [2]. US popularity is also increasing in academic fields as a complementary resource for learning anatomy and physical diagnosis, which students hold a high regard for [3]. In fact, a recent study implementing innovative strategies in education for undergraduate physical therapists found that including US in the curriculum was effective for learning anatomy, identifying musculoskeletal structures in other radiological images such as MRI, and preventing adverse effects during dry needling procedures by identifying high-risk structures located near the targeted muscles [4].

For research and clinical purposes, US is used for baseline assessments and monitoring changes in several musculoskeletal structures, supported by studies demonstrating the reliability and validity of these procedures [2,5,6,7,8]. For instance, B-mode US not only provides information about morphological and biomechanical characteristics (perimeter, cross-sectional area, aspect ratio, circularity, roundness, and volume) [9,10] but also histological aspects such as fat infiltration percentage and muscle quality, which can be estimated based on the pixels’ brightness [6].

The procedure to obtain this information starts with exporting the US images to 32-bit DICOM files (in a pure 256 gray-scale format). On this scale, connective tissues appear brighter than muscle fibers. As a result, brighter muscles indicate a higher presence of connective tissue [7]. Recent research has explored whether, by isolating a specific range of bright pixels, clinicians can accurately estimate the percentage of fatty infiltration in muscles. Given that the measurement is relative, this method could circumvent biases. These biases are associated with variations in acoustic impedance both between subjects and within them, or changes in gain settings [8].

One of the primary challenges faced when using B-mode US, especially in the imaging of larger muscles, is its restricted field of view. This limitation persists even when convex transducers are employed. Recognizing this challenge, researchers and developers have been working towards a solution known as panoramic US. Sometimes referred to as the extended field-of-view US, this technology aims to provide a broader and more comprehensive view of the structure’s area [11]. Panoramic US technology involves capturing a vast single image by automatically combining consecutive B-mode images as the transducer glides over the area of interest [12]. This method offers considerable potential for clinical assessments. For instance, it can aid in identifying histological alterations in chronic conditions, which are often linked with a graver prognosis and increased levels of disability and pain [13]. Additionally, it proves beneficial for monitoring patients who experience a loss in muscle mass, ensuring timely interventions and better patient care [14].

The rationale for conducting this study was firstly based on the limitations disclosed in a recent systematic review [15]. Although panoramic US seems to have an acceptable intra- and inter-examiner reliability for assessing muscle size and brightness, none of the studies included in the systematic review considered if the transducer glide may produce muscle deformation due to inadequate or variable gliding speed, pressure with the probe, or erratic gliding direction changes [15]. This issue was partially approached in a recent validity study [16] comparing the size, shape, and mean echo-intensity metrics of deep neck extensors between classic 2D images and extended field-of-view US images (as no muscle deformation is expected with the former). However, another important limitation was the absence of a second examiner to determine inter-examiner reliability estimates and the consideration of the examiners’ experience as a confounding factor. This consideration is of high importance since it could be a determinant factor for implementing this procedure in clinical practice.

From a clinical perspective, assessing the muscle size and shape of the quadriceps muscles is encouraged as sarcopenia and muscle atrophy are frequently found in many conditions such as spinal cord injury, chronic obstructive pulmonary disease, type 2 diabetes, and cancer, as well as in the elderly population [17], and are closely associated with poorer muscle function, morbidity, and mortality [18]. A recent study published by Ozturk et al. [19] described significant inverse correlations between the rectus femoris cross-sectional area assessed with US and the five-item sarcopenia questionnaire, being a recommended procedure for predicting sarcopenia accurately in elderly populations.

Considering the importance of not only muscle size or brightness but also muscle shape assessment in clinical practice [20], the objectives of this study were to analyze the inter-examiner reliability of muscle shape, size, and brightness descriptors in panoramic US imaging and determine whether the examiners’ experience plays a relevant role in imaging acquisition concordance.

## 2. Materials and Methods

### 2.1. Study Design

A single-group inter-examiner reliability study was conducted to analyze the concordance of muscle size, shape, and brightness measures (obtained with panoramic US) between two examiners (one experienced examiner with more than 10 years of US clinical experience in assessing musculoskeletal structures and more than 300 h of US training, and one new examiner with 1 year of clinical experience in musculoskeletal US and 20 h of training) in two different rooms to avoid communication. Images were analyzed by two independent raters (one experienced with more than 10 years of experience in musculoskeletal US and one novel).

The Research Randomizer website v.4.0 (http://www.randomizer.org/, accessed on 4 December 2023) was used for randomizing (1) the order of participants and leg side for imaging acquisition by each examiner and (2) the images that each rater had to measure.

This research adhered to the guidelines set out in the Declaration of Helsinki and received approval from the Local Ethics Committee (ID: 27/2022) before participant recruitment commenced. Furthermore, we complied with the STAndards for the Reporting of Diagnostic accuracy studies (STARD) checklist/guidelines [21]. Additionally, we observed the principles outlined in the Guidelines for Reporting Reliability and Agreement Studies (GRRAS) [22].

### 2.2. Participants

Between November 2021 and March 2022, local announcements were posted in a private university located in Madrid (Francisco de Vitoria University, Spain) to recruit a sample of healthy volunteers. To be eligible for participation in the study, volunteers had to read and sign an informed consent form and complete a health history questionnaire. Participants with current or ongoing neuromuscular diseases, musculoskeletal injuries involving the lower limb, previous history of surgery in the lower limb, intake of pharmacological drugs altering the muscle tone, or any other underlying medical condition were excluded from participation.

The minimum sample size required for this study was estimated following the recommendations of Walter et al. [23]. Considering a minimum acceptable reliability (ρ0) of ICC = 0.75 (as this is the lower cut-off for good reliability), an expected reliability (ρ1) of ICC = 0.90 was chosen based on previous reliability studies [15], setting a significance level of α = 0.05 and a power of 1 − β = 0.8. Considering 2 raters (k = 2) and an expected dropout rate of 10% due to the longitudinal nature of the study, at least 37 volunteers would be required for appropriate statistical power.

### 2.3. Panoramic Ultrasound Imaging Capture

For all imaging procedures, Alpinion eCubei8 US equipment was utilized (Alpinion Medical Systems Co., Ltd.; Seoul, Republic of Korea), paired with a linear transducer (3–12 MHz, E8-PB-L3-12T). We standardized the equipment settings for all scans: gain at 55 dB, dynamic range at 85, brightness set to 17, imaging depth at 4 cm, and a frequency of 12 MHz as described in previous studies following the same image analyses in order to compare the metrics obtained with classic B-mode (where there is no potential deformation) and panoramic US by an experienced operator [5,16]. The panoramic US images were processed automatically using the ultrasound device’s built-in stitching algorithm. This feature seamlessly combines multiple overlapping images captured during the scanning process. The algorithm selects control points and aligns them algorithmically without manual intervention, ensuring a consistent and accurate representation of the scanned area. This automated process simplifies the procedure and enhances the reliability of the resulting composite image.

The rectus femoris was targeted since, even if previous studies demonstrated acceptable panoramic US validity for the cross-sectional area assessment of this muscle [14], no brightness or shape metrics were considered. Thus, confirming the absence of muscle deformation during image acquisition would increase the strength of previous studies using this methodology, highlighting the clinical interest of this muscle in populations with neuromuscular conditions [17,24,25].

**Figure 1:**
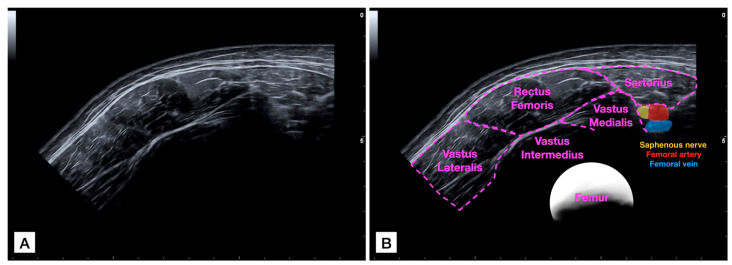
Raw panoramic ultrasound (US) imaging acquisition of the rectus femoris muscle (**A**) and image with identification of structures (**B**).

For acquiring the panoramic US image (Figure 1), the probe was placed in the medial and anterior aspect of the thigh at the mid-distance between the patellar base and the anterior inferior iliac spine to locate the sartorius muscle and visualize it in the lateral extreme of the image. Then, the probe was glided laterally (applying the minimum pressure possible) until it completely covered the rectus femoris muscle and the vastus lateralis was partially visualized. Gliding speed was controlled at 5 cm per second to try and apply a uniform light pressure during the path [16]. It was decided to set a constant speed to ensure that each scan is comparable, reducing variability introduced by different scanning speeds. In addition, faster speeds might result in blurred images or missed details, while slower speeds could lead to prolonged examination times without significant improvement in image quality. Both examiners underwent training sessions (practicing this protocol) for 30 min to familiarize themselves with the desired gliding speed.

### 2.4. Imaging Analysis

All acquired images underwent a methodical process before analysis. Initially, they were saved securely and then converted to DICOM format for standardized sharing and storage. Following the conversion, each image was coded to ensure a blind review and subsequently organized in a random sequence, ensuring the absence of any inherent sequence bias during the evaluation process. All images were then shared with the two appointed evaluators for thorough analysis. These procedures and the latter measurements were conducted using ImageJ software v.1.42 (National Institutes of Health, Bethesda, MD, USA).

To ensure consistency in analysis and interpretation, both evaluators were given precise and uniform instructions and special cautions were considered for avoiding communication to prevent consensus. They were directed to outline the rectus femoris meticulously, using the muscle’s internal fascia as the muscle contour selected in previous studies [14,16]. Once the rectus femoris was outlined, the same parameters describing the muscle size, shape, and histology analyzed in the validity study [16] were derived:

- Cross-sectional Area: This refers to the two-dimensional size of the muscle, quantified in cm^2^.
- Perimeter: Measured in cm, this is the total length of the contour of the muscle.
- Circularity: Determined using the formula 4π × Area/Perimeter^2^. A value of 1 in this metric signifies a perfect circle, indicating how close the muscle’s shape is to being circular.
- Aspect Ratio (AR): This is the quotient obtained by dividing the major axis (longest dimension) by the minor axis (shortest dimension) of the muscle’s shape.
- Roundness: This parameter is calculated using the formula 4 × Area/(π × major axis^2^), essentially being the inverse of the aspect ratio. It gauges how close the shape of the muscle is to a perfect circle.
- Solidity: This is a geometric measurement used to quantify the “fullness” of an object’s shape. It is calculated as the ratio of the area of the object to the area of its convex hull. The convex hull can be thought of as the smallest convex shape that completely encloses the object. A higher solidity value indicates that the object is closer to being completely convex, with fewer indentations or concavities. For example, a perfectly convex shape such as a circle has a solidity of 1, while shapes with indentations or irregular edges have lower solidity values.
- Mean Echo Intensity: A measure of the average brightness of the selected pixels, ranging on a scale from 0 (darkest) to 255 (brightest), indicating the relative brightness within the muscle image.

### 2.5. Statistical Analysis

All statistical calculations were conducted using SPSS software v.25 tailored for Mac OS (IBM, Armonk, NY, USA). The initial step in our analysis was to determine the normal distribution of our dataset; this was achieved through the Shapiro–Wilk test and histograms. Subsequently, we employed descriptive statistics to portray the demographic characteristics of the participants. These statistics provided a detailed breakdown, allowing for a comprehensive representation of the sample and offering insights segmented by gender.

For analyzing inter-examiner reliability, intraclass correlation coefficients (ICC_2,2_, being a two-way, mixed-model, and consistency type according to the classification proposed by Koo and Li [26]) were calculated using the scores obtained by both examiners for each metric. ICC_2,2_ scores were interpreted according to the cut-offs described in the literature (fair if ICC_2,2_ < 0.50, moderate if 0.50 < ICC_2,2_ < 0.75, good if 0.75 < ICC_2,2_ < 0.90, or excellent if 0.90 < ICC_2,2_) [26].

Additionally, we computed several statistical measures to further understand the precision and reliability of our evaluations. The Mean (or average) was calculated as Mean = $\frac{\Sigma \;US\;metric\;score\;}{Number\;of\;examiners}$, the Absolute Error was calculated as the absolute score difference between both examiners (AE = |Experienced Examiner − Novel Examiner|), the Standard Error of Measurement (SEM) was determined using the formula SEM = Standard Deviation of Absolute Error × √1 − ICC. The Minimal Detectable Change (MDC) was defined as MDC = 1.96 × SEM × √2, indicating the smallest discernible change that signifies a true difference beyond mere measurement error. The Coefficient of Absolute Error (CAE%), representing the relative variability of the dataset, was calculated as CAE% = Absolute Error/Mean Score × 100.

These metrics were assessed as, in reliability studies, the primary challenges are analyzing the degree of correlation and agreement between measurements (ICCs represent a ratio of true variance over true variance plus error variance while CAE% describes the relative variability of data in relation to the mean [26]) and distinguishing genuine changes in performance from random measurement errors (SEM offers a way to estimate the expected random variation in scores when no real change has occurred while MDC represents the smallest amount of change that needs to be observed for it to be considered a genuine change) [27].

Finally, in order to explore the potential influence of the examiners’ level of experience on the results, we conducted Student’s T-tests for independent samples, considering a *p*-value < 0.05 as indicative of statistical significance. Levene’s Tests for Equalicy of Variances were conducted to verify whether the variance of the scores across the groups defined by our examiners’ experience levels was statistically equivalent. A non-significant result in this test (*p* > 0.05) indicated that the assumption of homoscedasticity was met.

## 3. Results

From a total of 45 volunteers willing to participate in the study, six were excluded due to muscle relaxant intake (*n* = 2), muscle soreness (*n* = 1), and a history of anterior cruciate ligament reconstruction (*n* = 3). Therefore, 39 European individuals (no participants with a different ethnicity responded to the announcements) were included and 156 images (78 images per examiner) were acquired and analyzed. Demographic and anthropometric data of the sample detailed by sex are shown in Table 1. In general, males were significantly older, taller, heavier, and more overweight compared with females (all, *p* < 0.001). Regarding the anthropometric characteristics, both sexes showed comparable thigh length (*p* < 0.05) despite the significant girdle differences (*p* = 0.002).

Table 2 shows the reliability estimates of panoramic US acquisition by a novel and an experienced examiner for assessing the rectus femoris size, shape, and brightness. We found muscle area, perimeter, AR, circularity, roundness, and brightness to be excellently reliable (ICC_2,2_ > 0.9). On the other hand, solidity did not reach excellent estimates but showed good reliability (ICC_2,2_ = 0.869). The SEM estimates provide information about how repeated measures tend to be distributed around his or her “true” score, ranging from 0.00 (roundness, solidity, and circularity) to 0.51 (mean echo intensity). Regarding the MDC (this parameter is used to distinguish whether a change could be attributable to a real change instead of an instrument error), circularity, roundness, and solidity were shown to be the most sensitive metrics to detect real changes.

## 4. Discussion

To our knowledge, this is the first study analyzing whether panoramic US image acquisition alters muscle size, shape, or brightness due to potential transducer gliding differences between examiners, considering the experience of the examiners. In general, we found all parameters to be comparable between both examiners, even if both had different experiences with US scanning. In addition, agreement estimates were found to be excellent for metrics such as muscle area, perimeter, circularity, roundness, AR, and brightness, while they were merely good for solidity. The relatively poorer reliability estimates for solidity, which measures the muscle’s compactness, can be attributed to its inherent sensitivity to minor variations in the muscle’s contour. Factors such as small indentations, the number of measurement points during muscle contouring, protrusions, or imaging artifacts can significantly alter the solidity value. Such sensitivity can result in greater variability, especially when different examiners evaluate the same image or when a single examiner evaluates the image at different instances, consequently leading to lower ICCs. Improving image optimization (e.g., through enhancements in lateral and axial resolution, and dynamic range), could potentially increase the reliability of solidity measurements. Enhanced resolution would result in clearer and more defined muscle contours, reducing the impact of minor variations and artifacts that currently affect solidity values. This would lead to more consistent and accurate assessments, regardless of variations in examiner technique or image evaluation instances.

Although this is not the first study reporting morphology and histology by using panoramic US [11,14,15,16,17,28,29,30,31,32] or focusing on the rectus femoris muscle [14,15,17], this is the first study considering the inclusion of multiple shape descriptors (in addition to muscle quality and size estimates) when comparing the panoramic US images acquired by two examiners with different experiences.

For instance, Valera-Calero et al. [16] compared cervical multifidus and neck short rotator shape, size, and brightness metrics between classic 2D images and panoramic US, declaring that the rationale for studying these muscles was that they were small enough to be completely visualized in both modes. Although the obtained results were promising as no significant differences were found between modes and the concordance between methods was good to excellent, the most important limitation was the inclusion of a single experienced operator.

This research included a second novel operator to overcome this limitation, which is crucial for the clinical implementation of panoramic US. As described in the literature, novel examiners tend to obtain greater measurement errors due to uncontrolled variability in probe-to-skin force, inclination, and roll (especially if no probe force devices are used) [31]. Therefore, there is a potential risk of image deformation during the transducer glide and image acquisition. In addition, the targeted muscle was different as most of the panoramic US research focuses on the cross-sectional area, and shape descriptors and brightness are often missed [28]. Despite this background, there were no significant differences in the metrics obtained by both operators and the concordance between their measurements was excellent for all the metrics except solidity (which resulted in good concordance). In addition, the minimal detectable changes were small, supporting the accuracy of this procedure for follow-ups and ensuring that metric changes (if applicable) are real and not attributable to measurement errors. Hence, this research may enhance the evidence supporting the use of panoramic US for evaluating muscle size, shape, and brightness in large muscles, which were normally assessed with MRI or CT due to their large dimensions.

In contrast with MRI or CT (where imaging acquisition is automatic and is not operator-dependent), US depends directly on the examiner’s transducer handling [33,34,35,36,37]. One interesting finding is that the metric showing the worst MDC was the mean muscle echo intensity. The most reasonable hypothesis supporting this finding was provided by Ishida et al. [38], who described that probe tilts > 6° during rectus femoris scanning in healthy subjects result in significant mean echo intensity differences compared with a perpendicular positioning but no significant changes in muscle thickness [39], and also decrease the ability of US to detect real changes for both muscle thickness and brightness [38]. Therefore, it is essential to consider the transducer positioning and pressure to acquire reliable results.

Although it would be logical to think that experienced examiners may tend to be more uniform and consistent with the transducer pressure and angle, this association between experience in US imaging acquisition and reliability is not clear [40]. Although previous studies reported a large inter-examiner reliability variation depending on the examiners’ experience [41,42], several studies with highly detailed protocols demonstrated excellent reliability between novice and experienced examiners [42], even if the examiners are from different areas of expertise [43]. Therefore, detailed protocols setting reliable and specific references within the images is as important as transducer handling.

Since, in this study, we considered the scanning speed, structures to be firstly identified and secondly contoured, and US settings in addition to probe tilt and pressure, this may explain the excellent results obtained. However, the region assessed in this study may obtain better reliability estimates compared with other regions with larger paths or irregular curvatures [34], so developing auxiliary transducer devices may be considered in those regions with worse reliability estimates.

Based on these findings, we could suggest that panoramic US not only be used in cross-sectional designs due to the good-to-excellent reliability between examiners with different experience levels but also be used in longitudinal designs or clinical follow-ups since the capacity of panoramic US imaging to detect real changes (not attributable to measurement errors) was acceptable.

### Study Limitations and Future Directions

Despite our findings being promising, several limitations should be acknowledged. Firstly, we conducted this reliability study with only two examiners and a single US device. Further studies should consider the use of different devices and the inclusion of more examiners to corroborate these results. Secondly, the images acquired with panoramic US were not compared with a Gold Standard (e.g., MRI or CT). Thus, we only assessed a single muscle in healthy subjects. Considering the histologic changes in specific conditions, these results might not be applicable. Further research should consider other locations and further clinical populations. Finally, although both operators were asked to apply the minimum pressure possible with the probe, this aspect was not monitored. An interesting research line opened after conducting this research is to analyze differences among operators (novel and experienced) regarding the pressure exerted with the probe and analyze the correlation between the pressure and its variance with measurement errors.

## 5. Conclusions

The results obtained in this study suggest that panoramic US does not alter rectus femoris size, shape, or brightness based on the panoramic US images acquired by two different examiners with different levels of experience. The agreement between both examiners for assessing the cross-sectional area, perimeter, circularity, roundness, solidity, and mean echogenicity was excellent while the aspect ratio was good. Therefore, panoramic US could be used for research and clinical purposes in both cross-sectional and longitudinal designs to obtain reliable and accurate rectus femoris size, shape, and brightness US metrics in healthy subjects.

## Figures and Tables

**Table 1:** Baseline demographic characteristics (mean ± SD) of the sample and detailed by sex.

Baseline	Sample (*n* = 39)	Males (*n* = 27)	Females (*n* = 12)	Between-Sex Differences
Age (years)	25.1 ± 9.1	27.4 ± 10.3	23.2 ± 6.7	4.9 (2.6;7.2) *p* < 0.001
Height (m)	1.74 ± 0.09	1.78 ± 0.08	1.66 ± 0.06	0.11 (0.01;0.22) *p* < 0.001
Weight (kg)	71.9 ± 14.3	78.2 ± 12.5	57.8 ± 4.0	20.4 (6.0;34.8) *p* < 0.001
Body Mass Index (kg/m^2^)	23.3 ± 2.6	24.4 ± 2.2	20.7 ± 1.2	3.6 (1.0;6.3) *p* < 0.001
Thigh length (cm)	47.7 ± 2.5	48.3 ± 2.3	45.7 ± 2.8	2.6 (−0.6;5.7) *p* = 0.101
Thigh girdle (cm)	57.1 ± 5.3	58.6 ± 5.5	53.7 ± 2.1	4.8 (−1.4;11.2) *p* = 0.002

**Table 2:** Summary of examiners’ agreement for assessing rectus femoris size, shape, and brightness using extended field-of-view ultrasound imaging.

Baseline	Mean	Experienced Examiner	Novel Examiner	Absolute Error	ICC_2,2_ (95% CI)	SEM	MDC	CAE (%)
Muscle Size								
Area (cm^2^) *	10.54 ± 2.61	10.52 ± 2.64	10.57 ± 2.64	0.33 ± 0.26	0.993 (0.985;0.997)	0.02	0.06	2.46
Perimeter (cm) *	14.23 ± 2.45	14.26 ± 2.49	14.19 ± 2.45	0.23 ± 0.21	0.996 (0.991;0.998)	0.01	0.03	1.47
Muscle Shape								
Circularity (0–1) *	0.62 ± 0.06	0.63 ± 0.06	0.62 ± 0.05	0.02 ± 0.02	0.944 (0.874;0.975)	0.00	0.01	3.22
Aspect Ratio *	2.90 ± 0.46	2.91 ± 0.48	2.89 ± 0.44	0.13 ± 0.14	0.951 (0.891;0.978)	0.03	0.08	4.82
Roundness *	0.35 ± 0.06	0.35 ± 0.06	0.35 ± 0.05	0.02 ± 0.02	0.952 (0.893;0.979)	0.00	0.01	5.71
Solidity (0–1) *	0.98 ± 0.01	0.99 ± 0.01	0.98 ± 0.01	0.01 ± 0.01	0.869 (0.709;0.941)	0.00	0.01	1.02
Muscle Brightness								
Mean Echo-intensity (0–255) *	59.1 ± 15.6	58.3 ± 15.3	59.7 ± 16.1	2.82 ± 3.63	0.980 (0.956;0.991)	0.51	1.42	6.14

* No statistically significant differences between examiners were found (*p* > 0.05)

## Data Availability

All data derived from this study are presented in the text.

## References

[1] Whittaker J.L., Ellis R., Hodges P.W., OSullivan C., Hides J., Fernandez-Carnero S., Arias-Buria J.L., Teyhen D.S., Stokes M.J. (2019). Imaging with ultrasound in physical therapy: What is the PT’s scope of practice? A competency-based educational model and training recommendations. Br. J. Sport. Med..

[2] Valera-Calero J.A., Fernández-de-Las-Peñas C., Varol U., Ortega-Santiago R., Gallego-Sendarrubias G.M., Arias-Buría J.L. (2021). Ultrasound Imaging as a Visual Biofeedback Tool in Rehabilitation: An Updated Systematic Review. Int. J. Environ. Res. Public Health.

[3] So S., Patel R.M., Orebaugh S.L. (2017). Ultrasound imaging in medical student education: Impact on learning anatomy and physical diagnosis. Anat. Sci. Educ..

[4] Valera-Calero J.A., Navarro-Santana M.J., Fernández-de-Las-Peñas C., Varol U., López-de-Uralde-Villanueva I., Rodríguez-López E.S., Plaza-Manzano G. (2023). Inclusion of cross-sectional and radiological images for better understanding of musculoskeletal anatomy and decreasing the risk of adverse events during dry needling in undergraduate physiotherapy students. Anat. Sci. Educ..

[5] Fitze D.P., Franchi M.V., Peterhans L., Frey W.O., Spörri J. (2023). Reliability of panoramic ultrasound imaging and agreement with magnetic resonance imaging for the assessment of lumbar multifidus anatomical cross-sectional area. Sci. Rep..

[6] Fukumoto Y., Ikezoe T., Yamada Y., Tsukagoshi R., Nakamura M., Mori N., Kimura M., Ichihashi N. (2012). Skeletal muscle quality assessed from echo intensity is associated with muscle strength of middle-aged and elderly persons. Eur. J. Appl. Physiol..

[7] Stock M.S., Thompson B.J. (2021). Echo intensity as an indicator of skeletal muscle quality: Applications, methodology, and future directions. Eur. J. Appl. Physiol..

[8] Tanaka N.I., Ogawa M., Yoshiko A., Ando R., Akima H. (2017). Reliability of size and echo intensity of abdominal skeletal muscles using extended field-of-view ultrasound imaging. Eur. J. Appl. Physiol..

[9] Kadakia A., Zhang J., Yao X., Zhou Q., Heiferman M.J. (2023). Ultrasound in ocular oncology: Technical advances, clinical applications, and limitations. Exp. Biol. Med..

[10] Zhang J., Murgoitio-Esandi J., Qian X., Li R., Gong C., Nankali A., Zhou Q. (2022). High-Frequency Ultrasound Elastography to Assess the Nonlinear Elastic Properties of the Cornea and Ciliary Body. IEEE Trans. Ultrason. Ferroelectr. Freq. Control..

[11] Cleary C.J., Nabavizadeh O., Young K.L., Herda A.A. (2022). Skeletal muscle analysis of panoramic ultrasound is reliable across multiple raters. PLoS ONE.

[12] Starkoff B. (2014). Ultrasound physical principles in today’s technology. Australas. J. Ultrasound Med..

[13] Zhu D.C., Lin J.H., Xu J.J., Guo Q., Wang Y.H., Jiang C., Lu H.G., Wu Y.S. (2021). An assessment of morphological and pathological changes in paravertebral muscle degeneration using imaging and histological analysis: A cross-sectional study. BMC Musculoskelet. Disord..

[14] Scott J.M., Martin D.S., Ploutz-Snyder R., Matz T., Caine T., Downs M., Hackney K., Buxton R., Ryder J.W., Ploutz-Snyder L. (2017). Panoramic ultrasound: A novel and valid tool for monitoring change in muscle mass. J. Cachexia Sarcopenia Muscle.

[15] Valera-Calero J.A., Ojedo-Martín C., Fernández-de-Las-Peñas C., Cleland J.A., Arias-Buría J.L., Hervás-Pérez J.P. (2021). Reliability and Validity of Panoramic Ultrasound Imaging for Evaluating Muscular Quality and Morphology: A Systematic Review. Ultrasound Med. Biol..

[16] Valera-Calero J.A., Plaza-Manzano G., Ortega-Santiago R., Fernández-de-las-Peñas C., Varol U. (2023). Panoramic ultrasound imaging does not produce muscle morphology deformation during imaging acquisition: A validity study. Phys. Med..

[17] Sahinis C., Kellis E., Galanis N., Dafkou K., Ellinoudis A. (2020). Intra- and inter-muscular differences in the cross-sectional area of the quadriceps muscles assessed by extended field-of-view ultrasonography. Med. Ultrason..

[18] Choi K.M. (2016). Sarcopenia and sarcopenic obesity. Korean J. Intern. Med..

[19] Ozturk Y., Koca M., Burkuk S., Unsal P., Dikmeer A., Oytun M.G., Bas A.O., Kahyaoglu Z., Deniz O., Coteli S. (2022). The role of muscle ultrasound to predict sarcopenia. Nutrition.

[20] Pons C., Borotikar B., Garetier M., Burdin V., Ben Salem D., Lempereur M., Brochard S. (2018). Quantifying skeletal muscle volume and shape in humans using MRI: A systematic review of validity and reliability. PLoS ONE.

[21] Simera I., Moher D., Hoey J., Schulz K.F., Altman D.G. (2010). A catalogue of reporting guidelines for health research. Eur. J. Clin. Investig..

[22] Kottner J., Audige L., Brorson S., Donner A., Gajewski B.J., Hróbjartsson A., Roberts C., Shoukri M., Streiner D.L. (2011). Guidelines for Reporting Reliability and Agreement Studies (GRRAS) were proposed. Int. J. Nurs. Stud..

[23] Walter S.D., Eliasziw M., Donner A. (1998). Sample size and optimal designs for reliability studies. Stat. Med..

[24] Ando R., Nosaka K., Inami T., Tomita A., Watanabe K., Blazevich A.J., Akima H. (2016). Difference in fascicle behaviors between superficial and deep quadriceps muscles during isometric contractions. Muscle Nerve.

[25] Burton A.M., Stock M.S. (2018). Consistency of novel ultrasound equations for estimating percent intramuscular fat. Clin. Physiol. Funct. Imaging.

[26] Koo T.K., Li M.Y. (2016). A guideline of selecting and reporting intraclass correlation coefficients for reliability research. J. Chiropr. Med..

[27] Furlan L., Sterr A. (2018). The Applicability of Standard Error of Measurement and Minimal Detectable Change to Motor Learning Research-A Behavioral Study. Front. Hum. Neurosci..

[28] Adkins A.N., Murray W.M. (2020). Obtaining Quality Extended Field-of-View Ultrasound Images of Skeletal Muscle to Measure Muscle Fascicle Length. J. Vis. Exp..

[29] Rosenberg J.G., Ryan E.D., Sobolewski E.J., Scharville M.J., Thompson B.J., King G.E. (2014). Reliability of panoramic ultrasound imaging to simultaneously examine muscle size and quality of the medial gastrocnemius. Muscle Nerve.

[30] Jenkins N.D., Miller J.M., Buckner S.L., Cochrane K.C., Bergstrom H.C., Hill E.C., Smith C.M., Housh T.J., Cramer J.T. (2015). Test-Retest Reliability of Single Transverse versus Panoramic Ultrasound Imaging for Muscle Size and Echo Intensity of the Biceps Brachii. Ultrasound Med. Biol..

[31] Valera-Calero J.A., Gallego-Sendarrubias G.M., Fernández-de-Las-Peñas C., Cleland J.A., Ortega-Santiago R., Arias-Buría J.L. (2020). Panoramic Ultrasound Examination of Posterior Neck Extensors in Healthy Subjects: Intra-Examiner Reliability Study. Diagnostics.

[32] Kennedy V.L., Flavell C.A., Doma K. (2019). Intra-rater reliability of transversus abdominis measurement by a novice examiner: Comparison of “freehand” to “probe force device” method of real-time ultrasound imaging. Ultrasound.

[33] Snodgrass S.J., de Zoete R.M.J., Croker C., Yerrapothu M., Elliott J.M. (2019). Reliability of cervical muscle volume quantification using magnetic resonance imaging. Musculoskelet. Sci. Pract..

[34] Kuzu Ö., Aras B. (2022). Sonographic measurement of the neck extensor muscle thickness in patients with fibromyalgia. Musculoskelet. Sci. Pract..

[35] Pedler A., McMahon K., Galloway G., Durbridge G., Sterling M. (2018). Intramuscular fat is present in cervical multifidus but not soleus in patients with chronic whiplash associated disorders. PLoS ONE.

[36] Smith A.C., Albin S.R., Abbott R., Crawford R.J., Hoggarth M.A., Wasielewski M., Elliott J.M. (2020). Confirming the geography of fatty infiltration in the deep cervical extensor muscles in whiplash recovery. Sci. Rep..

[37] Walton J.M., Roberts N., Whitehouse G.H. (1997). Measurement of the quadriceps femoris muscle using magnetic resonance and ultrasound imaging. Br. J. Sport. Med..

[38] Ishida H., Suehiro T., Suzuki K., Yoneda T., Watanabe S. (2017). Influence of the ultrasound transducer tilt on muscle thickness and echo intensity of the rectus femoris muscle of healthy subjects. J. Phys. Ther. Sci..

[39] Ishida H., Suehiro T., Watanabe S. (2016). Influence of Inward Pressure of the Transducer on Thickness and Echo Intensity of the Rectus Femoris Muscle During Ultrasonography. Middle East J. Rehabil. Health Stud..

[40] Carr J.C., Gerstner G.R., Voskuil C.C., Harden J.E., Dunnick D., Badillo K.M., Pagan J.I., Harmon K.K., Girts R.M., Beausejour J.P. (2021). The Influence of Sonographer Experience on Skeletal Muscle Image Acquisition and Analysis. J. Funct. Morphol. Kinesiol..

[41] Cavaggion C., Navarro-Ledesma S., Luque-Suarez A., Juul-Kristensen B., Voogt L., Struyf F. (2023). Subacromial space measured by ultrasound imaging in asymptomatic subjects and patients with subacromial shoulder pain: An inter-rater reliability study. Physiother. Theory Pract..

[42] Fortin M., Rosenstein B., Levesque J., Nandlall N. (2021). Ultrasound Imaging Analysis of the Lumbar Multifidus Muscle Echo Intensity: Intra-Rater and Inter-Rater Reliability of a Novice and an Experienced Rater. Medicina.

[43] Chiaramonte R., Bonfiglio M., Castorina E.G., Antoci S.A.M. (2019). The primacy of ultrasound in the assessment of muscle architecture: Precision, accuracy, reliability of ultrasonography. Physiatrist, radiologist, general internist, and family practitioner’s experiences. Rev. Assoc. Med. Bras..

